# The largest viable aortic aneurysm

**DOI:** 10.1002/ccr3.3263

**Published:** 2020-08-26

**Authors:** Salwa A. Koubaissi, Salah Zein‐El‐Dine

**Affiliations:** ^1^ Pulmonary and Critical Care Division Department of Internal Medicine American University of Beirut Medical Center Beirut Lebanon

**Keywords:** aneurysm, aortic aneurysm, dacron, dissecting, postoperative complications

## Abstract

Time has allowed us to attain new therapeutic advances in both surgical and medical fields. Nevertheless, prolonging patients’ life expectancies by using these new techniques exposes physicians to challenging and exceptional medical presentations that, in the near past, were not possibly attainable and would not have naturally occurred.

## Graphical abstract

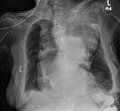

## 1 CASE PRESENTATION

**FIGURE 1:**
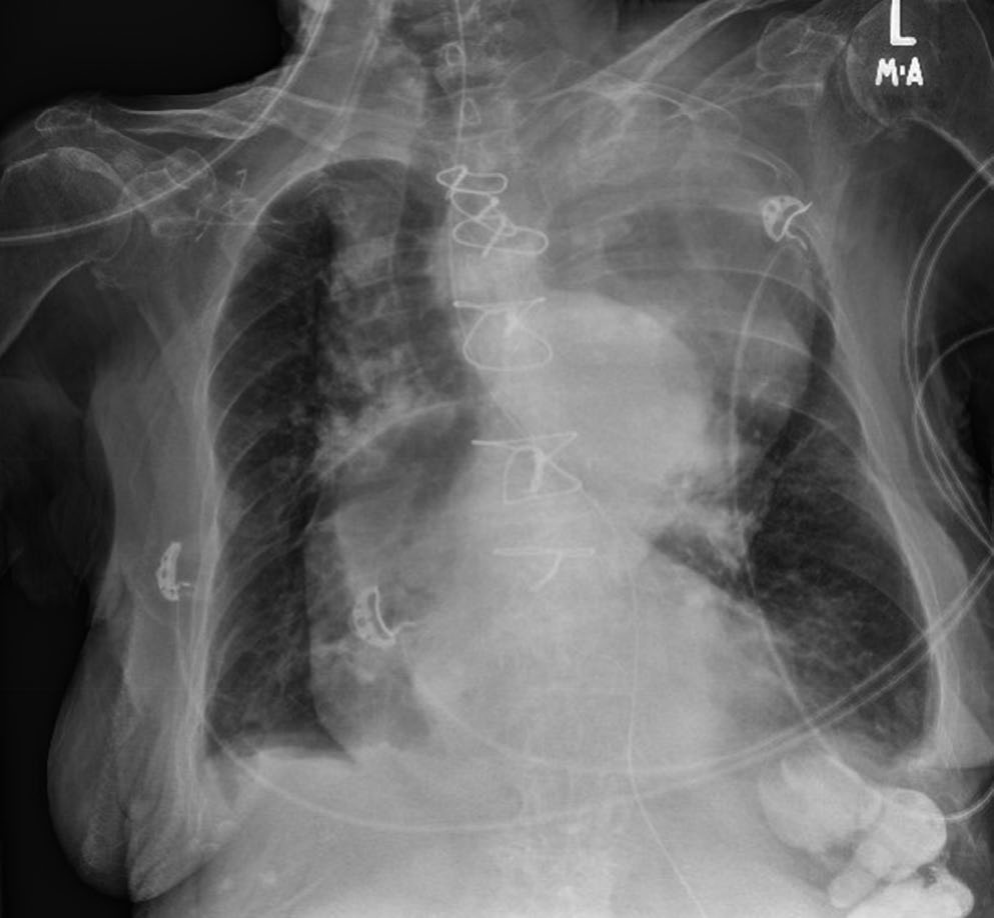
Large dissecting ascending aortic aneurysm occupying more than half of the mediastinal width

**FIGURE 2:**
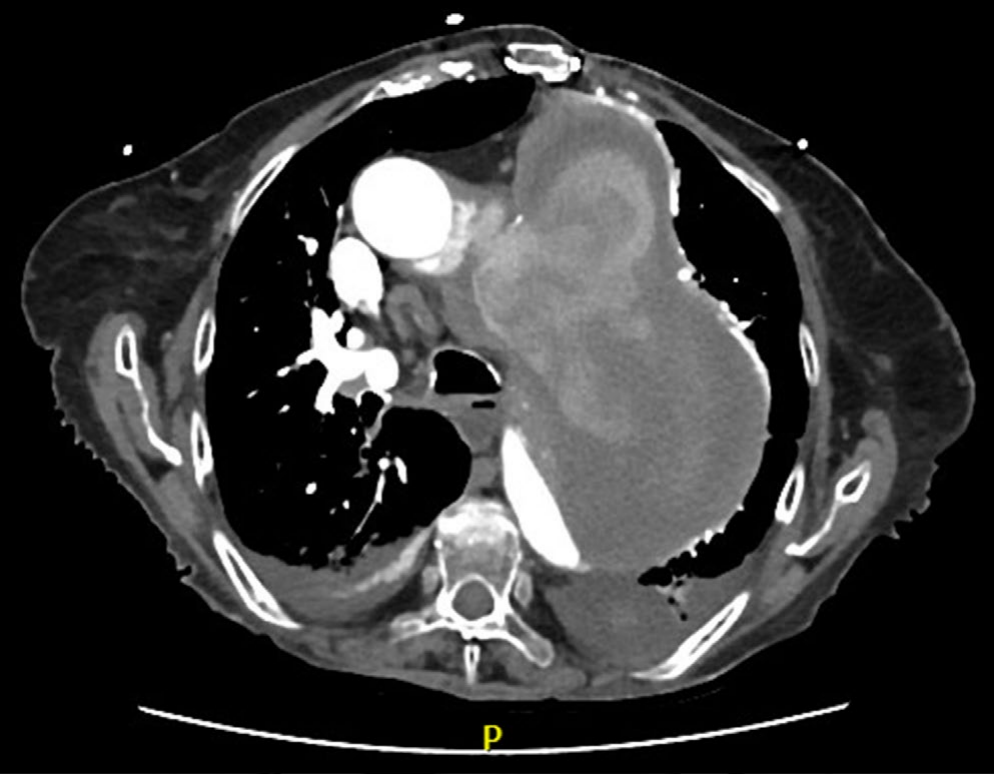
Sagittal view of the same chest X‐ray showing an 11 cm dissecting ascending aortic aneurysm with blood leakage into its false lumen

**FIGURE 3:**
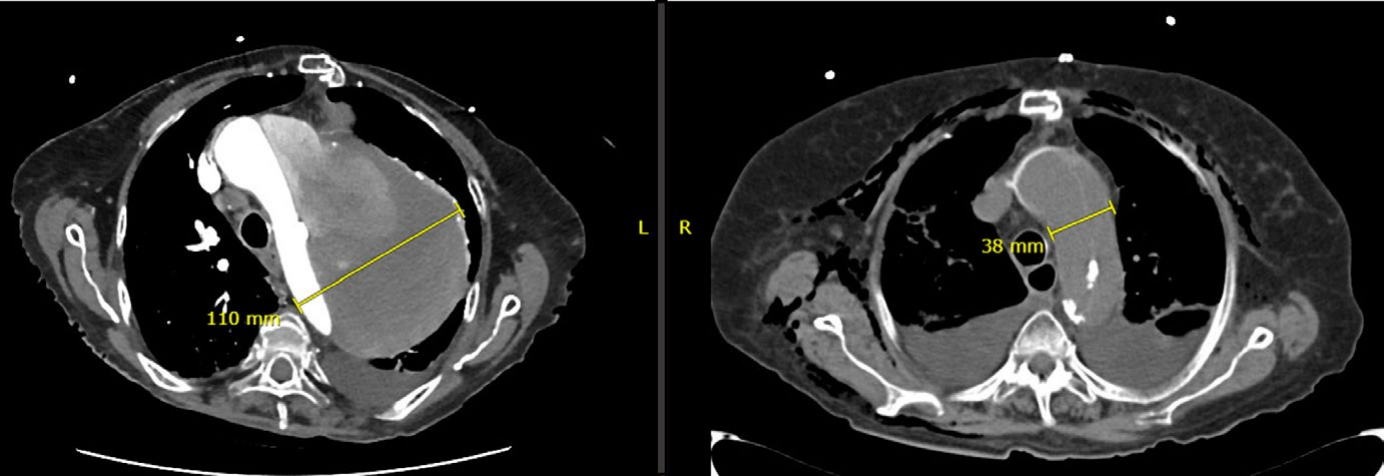
Degree of the anastomotic aortic aneurysm enlargement upon presentation (left) and in the immediate postoperative period (right), 7 y earlier

An 89‐year‐old woman, heavy smoker, with history of type A ascending aortic dissection status post‐replacement with a straight 32 mm Dacron conduit^1^ in 2012 presents for dyspnea and desaturation. Back during the operation 7 years earlier, the aortic tear was resected and the graft was beveled in with construction of an end‐to‐end anastomosis. Postoperatively, the ascending aorta measured 3.8 cm. A yearly follow‐up with chest computed tomography (CT) showed progressive formation of a fusiform aneurysmal sac involving the aortic arch and descending aorta and containing the Dacron graft. Its size was estimated to be 5.4 cm in 2013, 7 cm in 2015, and 8.8 cm in 2017. A new chest radiograph (Figure 1) and CT angiography done upon presentation showed a large dissected 11 cm aortic aneurysm, with blood leakage into its enlarging false lumen (Figure 2). A CT comparing the initial aortic arch diameter of 3.8 cm in the immediate postoperative period in 2012 to the current significantly enlarged aorta is shown in Figure 3. The patient was treated for findings of pneumonia, and a cardiothoracic consultation judged her current condition as “non‐surgical”. Unfortunately, she developed sudden acute hypotension during her stay that did not respond to medical management and passed away.

## CONFLICT OF INTEREST

None to be declared.

## AUTHOR CONTRIBUTIONS

SK: drafted submission and submitted case report for publication. SZ: performed literature review and assisted with drafting of submission.
